# The epidemiology of major depression among adults in Norway: an observational study on the concurrence between population surveys and registry data – a NCDNOR project

**DOI:** 10.1186/s12889-024-18754-w

**Published:** 2024-05-17

**Authors:** Jørgen G. Bramness, Vidar Hjellvik, Anne Høye, Martin Tesli, Marit Haram, Wenche Nystad, Steinar Krokstad

**Affiliations:** 1 Department of Alcohol, Tobacco and Drugs, Norwegian Institute of Public Health, P.O.Box 222, Oslo, 0213 Norway; 2 Norwegian National Advisory Unit on Concurrent Substance Abuse and Mental Health Disorders, Innlandet Hospital Trust, Brumunddal, Norway; 3 Department of Clinical Medicine, UiT The Arctic University of Norway, Tromsø, Norway; 4 Section for Clinical Addiction Research, Oslo University Hospital, Oslo, Norway; 5 Department of Chronic Diseases, Norwegian Institute of Public Health, Oslo, Norway; 6 Center for Clinical Documentation and Evaluation (SKDE), Tromsø, Norway; 7 Division of Mental Health and Substance Abuse, University Hospital of North Norway, Tromsø, Norway; 8 Department of Mental Health and Suicide, Norwegian Institute of Public Health, Oslo, Norway; 9 Division of Mental Health and Addiction, Oslo University Hospital, Oslo, Norway; 10 Department of Public Health and Nursing, Faculty of Medicine and Health Sciences, HUNT Research Centre, NTNU, Levanger, Norway; 11 Levanger Hospital, Nord-Trøndelag Hospital Trust, Levanger, Norway

## Abstract

**Background:**

Mental health problems, and major depression in particular, are important public health issues. Following trends in the prevalence of major depression is difficult because of the costs and complications of diagnostic interviews and general population self-report health surveys. Scandinavian countries, however, have several central, population-based health registries. We aimed to investigate how well these registries capture the epidemiology of major depression in the population.

**Methods:**

In two Norwegian regional surveys of general population health, each repeated after 10 years, responders were asked to report depressive symptoms using the Hopkins Symptom Checklist (HSCL) or the Hospital Anxiety and Depression Scale (HADS). Data were linked to three central health registries capturing contact with primary care, specialist care and prescriptions for antidepressants, to investigate how well these registries reflected self-reported depressive symptoms.

**Results:**

Most responders scored low on Hopkins Symptom Checklist (HSCL) and the Hospital Anxiety and Depression Scale (HADS), but 10% and 13%, respectively, scored above cut-off, with only minor changes between the two survey times. Females scored higher than males. Older people scored lower than younger, and a social gradient was visible. Around 12% of those who scored above the cut-off on either scale were recorded in the central health registries during the following year. This correlation was highest in primary care data, followed by prescription data and lowest in specialist care. Females were more often recorded in registries (*p* < 0.001), as were younger people (*p* < 0.001).

**Conclusions:**

There was a strong association between scores on screening for major depression in the general population surveys and being recorded in central health registries. There was a low sensitivity of these registries. and there was some variation in how sensitive the central health registries were in picking up depression, especially for males and older people. However, the stability of the measures over time suggests we may get an impression of the prevalence of major depression in the general population by using data from the central health registries. A combination of primary care data, prescription data and specialist care data have a higher sensitivity.

**Supplementary Information:**

The online version contains supplementary material available at 10.1186/s12889-024-18754-w.

## Introduction

Severe mental health disorders, major depression, and anxiety disorders account for a sizable and potentially increasing part of non-communicable diseases and form a disproportionately high burden of disease in all regions of the world. According to Global Health Estimates for the WHO European Region these disorders accounted for 29% of non-fatal disease burden (years lived with disability) and 15% of total disease burden (disability-adjusted life years; DALYs) [1]. However, these figures do not capture the full consequences of these disorders. They contributed heavily to as many as 141 000 deaths attributed to self-harm in the European Region in 2016 [2]. Furthermore, people with severe mental disorders and drug use disorders have a much higher average mortality compared to the general population, which translates to a reduction in life expectancy of 10–15 years [3]; these premature deaths are most commonly due to unrecognized and untreated physical health conditions [4, 5].

It is vital in the work on prevention and treatment of non-communicable diseases that we have reliable estimates of the occurrence of mental health disorders. Major depression is one of the most common disorders with a life-time prevalence of 5–17% [6]. The prevalence varies between groups: traditionally, more depressive symptoms are reported by females than by males [7–9], and more females than males are diagnosed with [10] and treated for [11] major depression, although this can vary [12]. Several studies have shown social gradients in depressive symptoms with lower socio-economic status (SES) being associated with more depressive symptoms [13, 14]. Still a study from Denmark found that higher socio-economic status predicts more use of antidepressants [15], while the reverse has been shown in Norway [16, 17]. It has also been debated whether the threshold for reporting mental health issues has changed over time [18]. There have been reports of increasing trends for reporting depressive symptoms, especially in females and at least in adolescents [19–21]. Furthermore, some studies have reported an increase in diagnosis [22] and treatment of major depression [23, 24], but this is not true for all studies [12, 25].

Unfortunately, data on mental health disorders are difficult to collect [26]. One approach is to perform population-based diagnostic interviews trying to capture the prevalence. However, such surveys are arduous, costly, and potentially have high attrition rates resulting in different forms of selection bias. Only a few such studies have been performed in Norway [8, 9, 23, 27].

An alternative approach is to conduct general population health surveys, using self-report questionnaires, rather than diagnostic tools. These are easier and cheaper to administer [28]. Although self-reporting of mental discomfort and depressive symptoms is not the same as having a mental health or depressive disorder [29], there are several validated self-report instruments for depressive symptoms with set cut-offs for major depression with established sensitivity and specificity. Two examples are the Hopkins Symptom Checklist (HSCL – referred to below as Hopkins) and the Hospital Anxiety and Depression Scale (HADS). Several studies have shown that they also tap into clinically valid information. Using Hopkins with a cut-off of 1.85 gives a sensitivity of 0.89 and a specificity of 0.98 for major depressive disorder [30]. Using HADS with a cut-off of 6 gives a sensitivity of 0.88 and a specificity of 0.70, the more optimal cut-off being 8 or 11 [31]. Although they cost less, such surveys are not performed in the whole population on a regular basis, so they are not ideal for ongoing estimates of prevalence.

An alternative, in Scandinavian countries, could be using national health registries and databases with nationwide coverage to provide estimates, as this information is already collected. Norway has several registries that could provide information about non-communicable diseases in general and major depression specifically. The Norwegian Control and Payment of Health Reimbursements Database, is an administrative register that covers treatment contacts in primary health care [32]. We refer to this below as the “primary care database”. the Norwegian Prescription Database (NorPD) covers prescriptions dispensed outside of institutions [33]. This is referred to below as “the prescription database”. The Norwegian Patient Registry (NPR) covers all treatment contacts in specialised health care [32], so this is referred to below as “the specialist care registry”. These registries offer readily available large data sets with full coverage and possibly less bias than surveys, but they are “shallow” in that they do not include much clinical detail or desired control variables [34]. Furthermore, the large discrepancy between symptoms reported and depression care received known from international [35] and national [36] research may imply that these registries do not represent the true morbidity in the population. Investigations have shown that only 36% of patients with major depressive disorder have been recorded for this in primary care and only 15% in specialised health care [36]. So, we need to find out how these registries may reflect the real illness levels in their target populations. Some groups may be better represented, and other groups less well.

In this study, we wanted to explore how well three of the central administrative/health registries in Norway – the primary care database, the prescription database, and the specialist care treatment registry – reflect the morbidity caused by major depression in the population. This was done by linking self-report of depressive symptoms in population-based health surveys to data from the health registries. We ask the following questions:

1. What is the level of self-reported depressive symptoms in population-based surveys, and does this level differ between age groups, sexes, or socioeconomic groups or over time?
2. What are the recodings of cases of depression found in population-based surveys in the central health registries, and does this differ between age groups, sexes or socioeconomic groups or over time?

## Materials and methods

### Health survey data

Data were retrieved from four different Norwegian population based health presented here by name (and year for data collection): two waves of the Tromsø Study, Tromsø6 (2007-08) and Tromsø7 (2015-16), and two waves of the Trøndelag Health Study, HUNT3 (2006-08) and HUNT4 (2017-19). All these surveys are comprehensive general population health surveys based in urban and rural areas of northern and central Norway targeting the resident adult population. Both Tromsø surveys included adults 40–70 years of age and both HUNT surveys included adults 20–79 years of age [37].

From all responders in the Tromsø6 and Tromsø7 surveys, the score on the Hopkins Symptom Checklist 10-item version was noted. HSCL-10 is a measure of psychological distress [38], and is a shorter version of the Hopkins checklist, which performs almost as well as the longer versions [30]. The HSCL-10 asks the respondent about symptoms related to anxiety and depression over the past week on a scale of 1 (not at all) to 4 (extremely). The mean score is calculated, producing a range of scores from 1 to 4 where higher score corresponds to more psychological distress. An average score ≥ 1.85 has commonly been considered a cut-off to identify cases [30]. HSCL-10 consists of two factors – depression and [39, 40] anxiety. For this study the five items covering depression were included. Only those responding to all five questions were included, no imputation was performed.

For all the responders in the two population surveys HUNT3 and HUNT4 responses for the Hospital Anxiety and Depression Scale (HADS) were recorded. HADS is a 14-item scale measuring self-reported anxiety and depression, where the scale goes from 0 to 3. There is a reliable two factor structure of the instrument [41], with items 2, 4, 6, 8, 10, 12 and 14 tapping depression (HADS-D). From these seven questions a sum score was calculated. Usually, three cut-offs are set for HADS-D: ≥5 for mild depression, ≥8 for moderate depression and ≥ 11 for severe depression [42]. To enable a comparable rate for depression with HSCL we used a HADS cut-off score of 7 or more as indicative of depression. Only those responding to all seven questions were included. No imputation was performed.

### Data from central health registries

The data from the four population-based surveys – Tromsø6, Tromsø7, HUNT3 and HUNT4 - were linked with data from three national databases and registries; the Norwegian Control and Payment of Health Reimbursements Database (the primary care database) 2006–2020, the Norwegian Prescription Database 2004–2020, and the Norwegian Patient Registry (specialist care registry) 2008–2020 using the Norwegian 11-digit unique person-identifier, encrypted. Codes for identification of outcome and their interpretation (wording) are given in Table 1. We looked for records in the registries 0-365 days after the date of the health survey symptom scores.

**Table 1:** Overview of the central health registries and diagnostic codes used

Register	Codes	Interpretation/wording
*Norwegian Control and Payment of Health Reimbursements Database (CPHR – the primary care database)*		
ICPC-2	P73, P76	Affective disorder, Depressive disorder
ICD-10	F32-F34	Depressive episode, recurrent depressive disorder, persistent mood (affective) disorder
*Norwegian Prescription Database (NorPD)*		
Drug ATC-codes	N06A*	Antidepressants
Reimbursement codes ICD-10	F32-F34, -F3	Affective disorders needing treatment, Affective disorders
Reimbursement codes ICPC-2	P73, P76, -73	
*Diagnosis recorded in Norwegian Patient Registry (NPR – the specialist care registry)*		
ICD-10	F32-F34	Depressive episode, recurrent depressive disorder, persistent mood (affective) disorder

* any digit after this, indicating all antidepressants

The primary care database is an administrative database that registers all claims from primary health care providers (doctors, psychologists, physiotherapists, etc.) in Norway from 2006 onwards. The database has full national coverage and all primary health care physicians send their claims to the Norwegian authorities. From the primary care database, we included all patients who were given an ICPC-2 diagnosis of a depressive disorder P73 or P76 or an ICD-10 diagnosis of F32-34 at least once. For the Tromsø6 and the HUNT3 surveys – because these were earlier - we only had 1 year of data available from the primary care database.

In the prescription database (available from 2004 onwards) we only registered those prescriptions for antidepressants (ATC-code N06A*) that included a reimbursement code for depression (any combination of ICD-10 codes -F3 and F32-F34 or ICPC-2 codes − 73, P73, and P76; Table 2), to avoid including prescriptions of antidepressants for other reasons. To ensure that these were, in fact, used for depression, we only included as outcome those who received *at least two* prescriptions between 90 and 365 days apart. For the 1-year window, at least one of the two prescriptions had to be within the window.

**Table 2:** Overview of the Norwegian population surveys included

Survey	Years performed	Age range	Screening instrument used	Number of invitees	Number (%) with complete data
Tromsø6	2007-08	40–79 years of age	5 items from HSCL-10	11,899	10,991 (92.4%)
Tromsø7	2015-16	40–79 years of age	5 items from HSCL-10	21,082	19,423 (92.1%)
HUNT3	2006-08	20–79 years of age	7 items from HADS	41,184	37,560 (91.2%)
HUNT4	2017-19	20–79 years of age	7 items from HADS	42,053	38,560 (91.7%)

Abbreviations: HSCL: Hopkin’s Symptom Checklist; HADS: Hospital Anxiety and Depression Scale; HUNT: Health Survey of Nord-Trøndelag County

The specialist care registry (NPR) covers all treatment of patients in specialised health care in Norway, with full national coverage since 2008. From the specialist care registry, we included all patients who were given an ICD-10 diagnosis of F32-F34 at least once.

### Background variables

Data on included ages, screening instruments for depressive symptoms, number of invitees and responders (and rates) are given in Table 2. Background variables included in the study were age, sex, and socio-economic status. Socio-economic status was measured by level of education and income by linking to data provided by Statistics Norway. Education was stratified into primary (12 years), secondary (15 years) and tertiary (≥ 16 years), and income into quartiles of household income per consumption unit, where the number of consumption units was computed according to the OECD modified scale which assigns a value of 1 to the household head, of 0.5 to each additional adult and of 0.3 to each child [43]. The quartiles were computed for the total Norwegian population per calendar year, sex, and 10-year age group (20–29, 30–39,…,70+).

### Statistical analysis

For the later surveys (Tromsø7 and HUNT4), we computed relative risks (RRs) for being recorded in one of the three health registries 0-365 days after the survey date, using uni- and multivariate Poisson regressions with robust variance estimates. The variables included in the regression models were the psychological distress HSCL/HADS score (above limit yes/no), age (continuous, per 10-year increment), sex (male/female), education (low/medium/high), income quartile (q1, q2, q3, q4), the last category being the reference for each categorical variable. Analyses were performed using R version 4.0.3.

## Results

### Response to general health survey questions on depressive symptoms

**Fig. 1:**
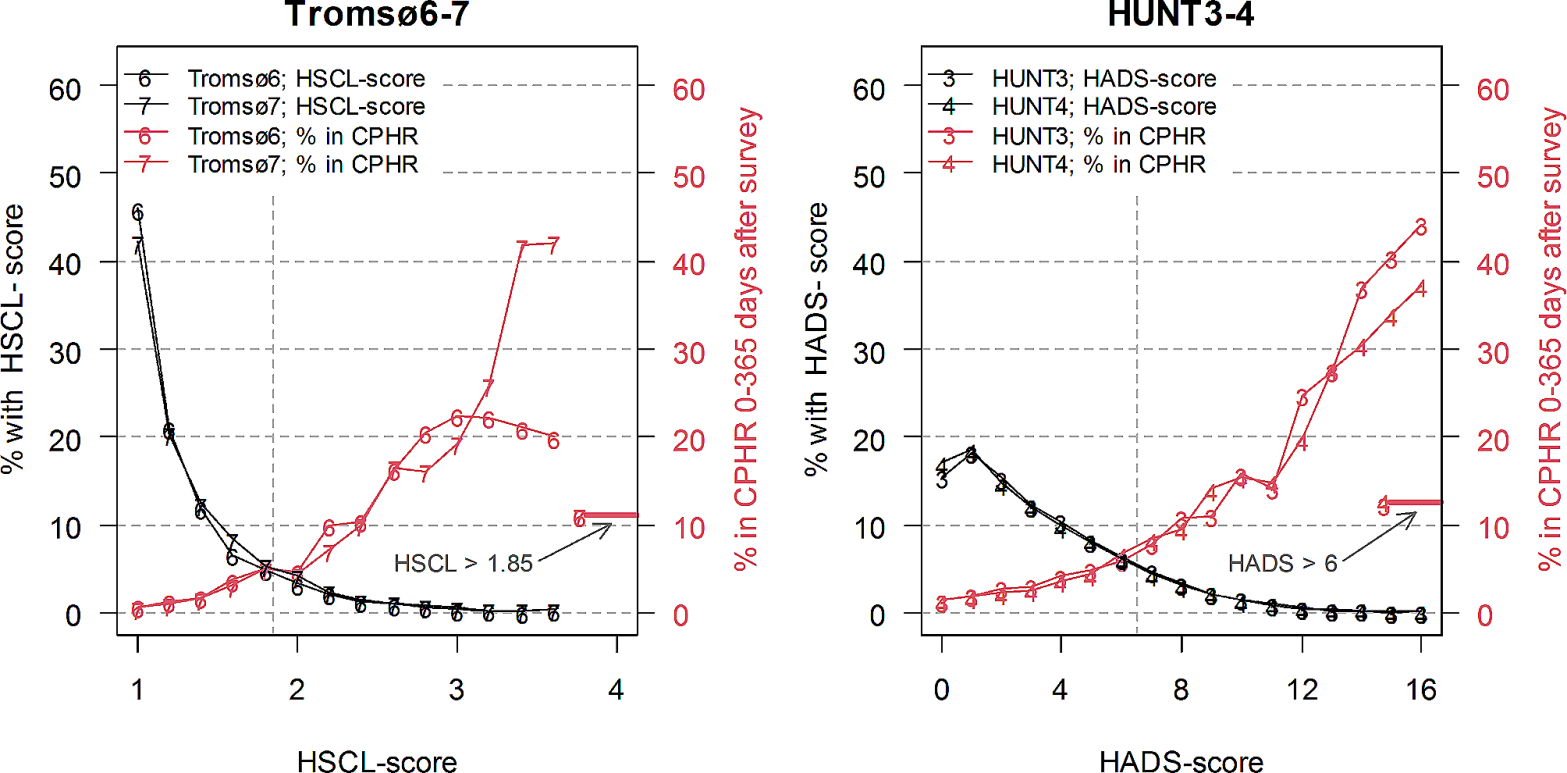
The distribution of scores (in percentage of the whole sample) on HSCL (average of 5 depression items, with a cut-off at an average score of > 1.85) in Tromsø6 and 7 (left panel) and HADS-D (sum of 7 depression items, with a cut-off score sum score of 7 or more) in HUNT3 and 4 (right panel). Both panels also show the share of responders that are found in the CPHR (primary care database) with an ICPC-2 diagnosis P73 or P76 or an ICD-10 diagnosis F32, F33 or F34 within 1 year after the survey. Black lines represent the earliest surveys, red lines the latest surveys. The included age span in Tromsø is 40–79 years, and in HUNT 20–79 years

Figure 1 shows that most people scored low on both the Tromsø (panel A) and the HUNT (panel B) population surveys, with minor changes between the two time points at which each of these surveys was repeated. The distribution of both Hopkins HCSL-10 (Tromsø6 and 7) and HADS (HUNT3 and 4) scores were skewed heavily towards the left, or to lower scores. This was most obvious in the Tromsø surveys which used the Hopkins HCSL-10 scoring instrument. In Table 3 we see an increase in the number who scored above cut-off from the Tromsø6 (2007-08) to the Tromsø7 (2015-16) survey (9.6–11.4%; change of 1.8 [95% CI 1.0, 2.5]), while the number of responders scoring above cut-off showed a slight decrease from HUNT3 (2006-08) to HUNT4 (2017-19) (from 13.9 to 13.5%; change of -0.4; [-0.9, 0.1]). For the Tromsø studies, there was an overweight of females scoring above cut-off, while for the HUNT surveys there was an overweight of males. Table 3 (and supplementary figure S1) shows the age distribution of respondents who reported depressive symptoms above cut-off. In the HUNT Study, the score versus age was reversed with the elderly having the highest share above cut-off in the 2006-08 study, but the youngest in the 2017-19 survey. In the Tromsø surveys, there was a similar trend over time but less pronounced. There was a strong social gradient for reporting depressive symptoms above cut-off with those who had lower socio-economic status (both education and income) more often scoring above cut-off. This gradient was more pronounced in the HUNT surveys and when using income – rather than education - as determinant of socio-economic status.

**Table 3:** Number and proportion of those scoring above cut-off in the population surveys on HSCL or HADS and the share of these that are found in different central health registries 0-365 days after symptom scores

				Early surveys (Tromsø6 and HUNT3)		Late surveys (Tromsø7 and HUNT4)					
Study area	Group							Represented in (%)			
			N ^a^	Scoring above cut-off (%)	Recorded in CPHR (%)	N*	Scoring above cut-off (%)	CPHR	NorPD	NPR	Any
Tromsø	All	All	10,991	9.6	11.0	19,423	11.4	11.2	8.7	3.9	16.1
	Sex	Female	5662	11.8	12.4	10,140	13.3	12.4	9.2	3.9	17.4
		Male	5329	7.3	8.7	9283	9.3	9.3	7.8	3.9	14.2
	Age (years)	40–49	3262	10.4	12.7	5829	13.2	13.4	8.6	5.2	18.2
		50–59	2322	10.9	13.0	5926	13.9	12.5	8.1	3.8	16.6
		60–69	3775	8.5	9.4	5067	9.1	8.0	9.1	3.0	13.4
		70–79	1632	9.0	7.5	2601	6.1	-	10.7	-	11.3
	Education	Low	2433	12.4	10.6	2968	13.6	8.9	12.9	2.5	17.4
		Medium	4554	9.8	9.2	7048	11.5	9.7	6.7	3.0	13.5
		High	3992	7.8	14.2	9356	10.6	13.3	8.7	5.2	17.8
	Income	q1	1892	16.1	12.8	2639	20.6	13.8	12.1	5.3	21.1
		q2	2881	10.0	12.5	5038	12.9	12.7	9.4	4.3	17.9
		q3	3219	7.9	9.1	6044	9.4	10.7	6.8	3.5	13.8
		q4	2981	7.0	9.1	5679	7.9	6.3	5.8	2.2	10.3
HUNT	All	All	37,560	13.9	12.4	38,560	13.5	12.7	12.8	3.7	20.5
	Sex	Female	20,969	12.5	16.3	22,034	12.6	16.4	16.0	4.7	25.7
		Male	16,591	15.6	8.5	16,526	14.6	8.6	9.2	2.5	14.6
	Age (years)	20–29	2655	7.2	19.4	3532	15.0	17.0	11.0	6.4	21.6
		30–39	4682	9.5	18.9	4413	14.8	18.8	10.2	6.7	22.3
		40–49	7612	12.3	15.6	6185	13.7	15.8	11.7	5.1	22.0
		50–59	9149	14.6	14.5	8214	12.9	12.8	12.4	4.0	20.8
		60–69	8501	15.6	9.0	9074	12.1	10.8	14.2	1.9	19.8
		70–79	4961	19.6	6.7	7142	14.0	5.9	15.5	0.7	18.1
	Education	Low	7368	19.8	11.7	5181	19.3	12.1	14.5	3.3	22.5
		Medium	18,898	14.2	12.0	18,219	14.0	10.8	12.6	2.5	18.8
		High	11,244	9.5	14.6	15,051	10.6	16.4	12.4	5.7	22.3
	Income	q1	8873	17.9	14.9	6612	20.5	15.0	14.8	4.3	24.4
		q2	11,852	13.9	11.8	11,489	14.3	12.7	13.7	3.4	21.4
		q3	10,067	12.2	10.7	11,614	11.5	11.6	10.7	3.8	17.9
		q4	6700	10.7	11.5	8802	9.6	11.0	11.4	3.1	17.0

Abbreviations: HSCL: Hopkin’s Symptom Checklist; HADS: Hospital Anxiety and Depression Scale; HUNT: Health Survey of Nord-Trøndelag County; CPHR: Norwegian Control and Payment of Health Reimbursements Database; NorPD: Norwegian Prescription Database; NPR: Norwegian Patient Registry

a) N with missing education in Tromsø6/7, HUNT3/4: 15, 55, 52, 115; N with missing income in Tromsø6/7, HUNT3/4: 21, 32, 97, 88

### Recordings in central health registries

Around 12% of those who scored above cut-off in either the Tromsø or the HUNT population surveys were found in the primary care database. This was the case for both the early and later surveys (Table 3). The rate of being recorded in the primary care database was closely and positively related to scores on the Hopkins and HADS scales (Fig. 1). There was no difference in the proportion recorded in the primary care database between the first survey and the second in either study, in the unadjusted or adjusted analysis (Table 4). More females than males were found in the primary care database, with only marginal developments over time. The sex difference was larger in the HUNT studies. There was an inverted age gradient of similar magnitude in the Tromsø and HUNT surveys. The higher the age, the lower the proportion recorded in the primary care database (Table 5). This was maintained over the two waves (supplementary figure S1).

**Table 4:** Relative risk of being recorded in CPHR (the primary care database) 0-365 days after symptom scores in later study compared to earlier study for those scoring above cut-off

		Found in CPHR (unadjusted)			Found in CPHR (adjusted ^a^)		
	Ref	RR	95%CI	p-value	RR	95%CI	p-value
**Tromsø**							
Later study	Earlier study = 1 (ref)	0.99	(0.80–1.22)	0.927	1.12	(0.91–1.37)	0.289
**HUNT**							
Later study	Earlier study = 1 (ref)	0.98	(0.88–1.08)	0.635	1.11	(1.00-1.22)	0.050

Abbreviations: CPHR: Norwegian Control and Payment of Health Reimbursements Database; RR: Relative risk; HUNT: Health Survey of Nord-Trøndelag County

a) adjusted for HSCL/HADS score (continuous), age (continuous; 40–79 years of age in Tromsø and 20–79 years of age in HUNT), sex, education (3 levels), and income quartile

**Table 5:** Binary poisson regression analysis for the risk of being recorded in central health registries (CPHR, NorPD, NPR or any). Data are given as relative risk (RR) and with a p-value. 95% confidence intervals for the RR can be found in supplementary table S1

		Found in central health registries (unadjusted)								Found in central health registries (adjusted ^a^)							
		CPHR		NorPD		NPR		Any		CPHR		NorPD		NPR		Any	
	Ref.	RR	95% CI	RR	95% CI	RR	95% CI	RR	95% CI	RR	95% CI	RR	95% CI	RR	95% CI	RR	95% CI
**Tromsø7**																	
HSCL (Above limit)	No	8.46	(7,10–10,07)	6.14	(5,11 − 7,38)	11.86	(8,52 − 16,52)	7.23	(6,30 − 8,29)	6.79	(5,64 − 8,16)	5.48	(4,53 − 6,64)	10.10	(7,17 − 14,23)	6.10	(5,29 − 7,04)
Age (10 years)	Cont.	0.68	(0,62 − 0,74)	0.98	(0,90 − 1,07)	0.74	(0,62 − 0,88)	0.81	(0,76 − 0,87)	0.77	(0,69 − 0,84)	1.10	(1,00–1,20)	0.89	(0,74 − 1,08)	0.91	(0,85 − 0,98)
Sex	Male	1.83	(1,52 − 2,21)	1.69	(1,39 − 2,05)	1.72	(1,22 − 2,42)	1.69	(1,46 − 1,96)	1.56	(1,30 − 1,89)	1.48	(1,21 − 1,79)	1.42	(1,00–2,01)	1.47	(1,27 − 1,70)
Education (medium)	High	0.83	(0,68 − 1,02)	1.02	(0,82 − 1,26)	0.66	(0,45 − 0,95)	0.90	(0,77 − 1,06)	0.82	(0,67 − 1,00)	0.89	(0,71 − 1,10)	0.60	(0,41 − 0,88)	0.84	(0,71 − 0,99)
Education (low)	High	0.91	(0,70 − 1,18)	1.54	(1,21 − 1,97)	0.72	(0,44 − 1,19)	1.23	(1,01–1,49)	0.78	(0,60 − 1,02)	1.15	(0,90 − 1,47)	0.57	(0,34 − 0,95)	0.99	(0,82 − 1,21)
Income, q3	q4	1.42	(1,06 − 1,90)	1.34	(1,00–1,79)	1.51	(0,90 − 2,54)	1.39	(1,11 − 1,75)	1.28	(0,96 − 1,71)	1.31	(0,98 − 1,76)	1.41	(0,84 − 2,36)	1.31	(1,05 − 1,64)
Income, q2	q4	2.35	(1,78 − 3,09)	2.08	(1,57 − 2,74)	2.21	(1,34 − 3,64)	2.21	(1,78 − 2,74)	1.91	(1,45 − 2,51)	1.83	(1,37 − 2,42)	1.80	(1,09 − 2,97)	1.86	(1,50 − 2,31)
Income, q1	q4	3.84	(2,90 − 5,08)	3.14	(2,36 − 4,19)	3.65	(2,18 − 6,10)	3.47	(2,79 − 4,33)	2.52	(1,88 − 3,37)	2.20	(1,63 − 2,98)	2.42	(1,43 − 4,10)	2.35	(1,87 − 2,95)
**HUNT4**																	
HADS (above limit)	No	4.66	(4,24 − 5,13)	3.56	(3,25 − 3,89)	6.90	(5,64 − 8,45)	3.82	(3,56 − 4,10)	4.54	(4,12 − 5,01)	3.43	(3,13 − 3,76)	6.82	(5,54 − 8,39)	3.68	(3,42 − 3,95)
Age (10 years)	Cont.	0.82	(0,80 − 0,85)	1.14	(1,10 − 1,17)	0.71	(0,67 − 0,76)	0.99	(0,97 − 1,01)	0.85	(0,83 − 0,88)	1.15	(1,12 − 1,18)	0.75	(0,70 − 0,80)	1.01	(0,99 − 1,03)
Sex	Male	1.89	(1,69 − 2,10)	1.86	(1,68 − 2,05)	1.77	(1,41 − 2,21)	1.85	(1,71 − 2,00)	1.86	(1,67 − 2,07)	2.03	(1,84 − 2,23)	1.63	(1,30 − 2,05)	1.94	(1,79 − 2,09)
Education (medium)	High	0.84	(0,76 − 0,94)	1.32	(1,20 − 1,47)	0.59	(0,47 − 0,74)	1.09	(1,01–1,18)	0.86	(0,77 − 0,95)	1.15	(1,03 − 1,28)	0.63	(0,50 − 0,79)	1.02	(0,94 − 1,11)
Education (low)	High	1.09	(0,94 − 1,26)	1.99	(1,76 − 2,26)	0.95	(0,71 − 1,28)	1.57	(1,42 − 1,74)	0.89	(0,77 − 1,04)	1.42	(1,24 − 1,62)	0.79	(0,58 − 1,08)	1.21	(1,09 − 1,34)
Income, q3	q4	1.16	(0,99 − 1,35)	1.12	(0,97 − 1,28)	1.21	(0,88 − 1,65)	1.15	(1,02 − 1,28)	1.13	(0,97 − 1,31)	1.08	(0,94 − 1,24)	1.17	(0,86 − 1,59)	1.11	(1,00–1,25)
Income, q2	q4	1.58	(1,37 − 1,83)	1.47	(1,29 − 1,68)	1.33	(0,98 − 1,81)	1.54	(1,39 − 1,72)	1.45	(1,25 − 1,68)	1.32	(1,16 − 1,51)	1.19	(0,88 − 1,62)	1.40	(1,26 − 1,56)
Income, q1	q4	2.07	(1,77 − 2,41)	2.05	(1,79 − 2,36)	1.91	(1,39 − 2,63)	2.09	(1,87 − 2,33)	1.68	(1,44 − 1,96)	1.63	(1,41 − 1,88)	1.48	(1,08 − 2,05)	1.68	(1,50 − 1,88)

Abbreviations: CPHR: Norwegian Control and Payment of Health Reimbursements Database (primary care database); NorPD: Norwegian Prescription Database; NPR: Norwegian Patient Registry (specialist care database); RR: Relative risk; HSCL: Hopkin’s Symptom Checklist; HADS: Hospital Anxiety and Depression Scale; q: quartile

a) adjusted for HSCL/HADS score, age (40–79 years of age in Tromsø and 20–79 years of age in HUNT), sex, educational level and income

Overall, above cut-off responders were recorded more often in the primary care database than in the prescription database, and least often in the specialist care registry (Table 3). More responders from the later HUNT4 survey who scored above the cut-off were found in at least one of the registries after one year than responders with scores above cut-off in the later Tromsø7 survey (20.5% vs. 16.1%; difference of 4.4 [2.5, 6.3]). This difference was mostly explained by more recordings in the prescription database, where 50% more of those above cut-off were treated with antidepressants in the HUNT4 survey than the Tromsø7 survey.

Scoring above cut-off on the Hopkins scale (the Tromsø surveys) was associated with more than 6 times the risk of being recorded in one of the central health registries, ranging from about 6 times risk for being given a diagnosis in primary health care or receiving a prescription for an antidepressant to a more than 10 times risk of being treated for depression in specialist care (Table 5). Scoring above cut off on the HADS-scale (HUNT surveys) showed similar patterns of increase but of a lower magnitude.

Females who scored above the cut-off in the population surveys were more often recorded in the central health registries than males (*p* < 0.001) (Tables 3 and 5 and S1). This difference was more pronounced in HUNT4 than in Tromsø7 for registration in any of the central health registries within one year, and also in each of the single registries. An exception was a similar rate of recordings for both sexes in the prescriptions database in Tromsø7 (3.9%).

In Tromsø7 and HUNT4, the likelihood of receiving help for depression in primary care (CPHR) and/or specialist care (NPR) decreased with increasing age, while the likelihood of drug treatment increased with increasing age (Table 5 and S1).

In Tromsø7 and HUNT4, the likelihood of receiving primary and/or specialist care for depression increased with increasing educational level, while the likelihood of drug treatment decreased with increasing education. With regard to income, higher income was associated with depression care and drug treatment (Tables 3 and 5).

The specificity of the central health registries for major depression seemed to be high (supplementary table S2), with values above 0.92 for all groups and studies. The sensitivity of the health registries was, however, low with values ranging from very low (0.01–0.07) in the specialist care registry to low (0.08–0.19) in the primary care database. The highest sensitivity was found when combining all three registries, which captured 11–26% of the major depression, the lowest values among older males in the Tromsø7 and the highest sensitivity among younger females in the HUNT4.

## Discussion

This study aimed to investigate how well central health registries – covering primary and secondary health care and a prescription database – reflected self-reported depressive symptoms in the general population. Ten to 13% of the responders scored above cut-off for depression on the screening instruments in the general population, with an overweight of females and young respondents. About 12% of those who scored above cut-off were recorded in the central health registries, more often in the primary care database than in the prescription database and least often in the specialist care registry. In the registries, females and younger people were more often represented. However, the stable proportion of self-reported cases which are also found in the central health registries indicates that these registries are well suited to following the epidemiology of major depression in the population over time.

### Depression prevalence in the population studies

We investigated depressive symptoms in two different general population studies in Norway, which were each carried out at two different time points. We found that the average depression symptom score remained constant, with approximately 12% of the population scoring above the set cut-off. Females scored higher than males when using the Hopkins scale (HSCL-10), but not with HADS. There was a strong age gradient, with fewer older people having high scores in either survey and at both times. The exception was the inverse relationship in the earlier HUNT3 survey, which may indicate a changed association between age and depression over time [21]. There was also a strong social gradient in the scoring of depressive symptoms, where those with lower socio-economic status scored higher than those with higher socio-economic status.

Eleven per cent of the population scored above cut-off for HCSL-10 and 14% above cut-off for HADS. With the known specificities of HSCL and HADS of 0.98 and 0.70 [30, 31, 44], respectively, and with a sensitivity of 0.88–0.89, this would indicate a point prevalence of 10–11% of the population had major depression. This is very close to the population-based surveys conducted previously in Norway [36, 45] and in other countries [40, 46, 47].

A higher female score on the Hopkins scale (HSCL-10) [48] and higher depression prevalence [7] has been found in many studies. The lack of sex difference in the HADS scale may be because the items included in HADS are more weighted towards the psychological aspects and less for the physical aspects of depression [49], moderating the sex difference [50].

An earlier HUNT publication showed that the prevalence of depressive symptoms increased with age in the 1990s [51]. In data from HUNT3, this distribution is still present, while in both surveys from Tromsø and the later HUNT4 survey this age gradient had changed significantly in the direction of a greater prevalence of depressive symptoms in young adults, reducing into adult age and lowest in the elderly [21]. Again, the psychometric properties of HSCL vs. HADS may have influenced the results in the Tromsø surveys vs. HUNT4, but not the difference found between HUNT3 and HUNT4. It seems to be a new trend that younger people report more symptoms of mental distress, and this finding is replicated in many newer surveys [52], but we do not know for certain how well these reports reflect a true increase in depressive symptoms.

Lastly, irrespective of how socio-economic status was measured, there was a significant social gradient with lower socio-economic groups reporting more depressive symptoms. This is a common finding across different studies [53, 54]. Traditionally Norway is a country with low economic differences, but a slight increase in these differences may be an important finding [55].

### Recordings in central health registries

There was a clear correlation between the increasing depression scores in the general population surveys and increased recordings in central health registries. This dose-response relationship shows the validity of using central health registries to monitor changes in trends of major depression in the population. As would be expected, more people were recorded in the primary care registry than in the prescription database, and fewest in the specialist care registry. Being recorded in any of the three health registries captured substantially more than any of the health registries alone. The specificity of the central health registries was overall very high, but the sensitivity of the registries was low, indicating that they do not reveal the true prevalence of major depression in the population.

The figures for recordings in central health registries the following year were 11–12% in the primary care database and around 4% in the prescriptions database. A twin study based on diagnostic interviews and national registry data concluded that 6.9% and 2.8% of the patients with major depression were also found in primary care and secondary care registries, respectively, over a period of approximately 3 years [36]. In our study, we included a slightly wider range of diagnoses from the primary care and prescription databases, which may have contributed to the higher numbers. Also, we used screening instruments which are not designed for diagnostic purposes. The known sensitivities and specificities of HSCL and HADS for major depression give a positive predictive value of scoring above cut-off as low as 30% [30, 31, 44]. Only if we were to set cut-off as high as 3 on HSCL or 14 on HADS (resulting in a dramatically reduced sensitivity) [30] would we get a treatment frequency comparable to that found in the diagnostic based population surveys [36]. The falling proportion of records from the primary care registry to the prescription registry and lastly to the specialist care registry appears natural, as many people with depression are followed up and treated in primary health care [12] and not everyone with depressive symptoms is treated with drugs. Even fewer are referred to specialist care. The exception here is the HUNT4 Survey where more people were treated with antidepressants than were given a diagnosis of depression in primary care. Even if we have information about the indication for prescribing, we may not be able to exclude all those receiving antidepressant prescriptions for other indications, such as anxiety, insomnia, or anorexia [56].

Females were more often recorded than males in all central health registries. This is similar to studies which have found that females more often seek help for mental health problems [57], and are more often prescribed antidepressants in general [11], even if results are more mixed for antidepressants prescribed for major depression [17]. This may still be of concern because of the negative health consequences, such as suicide, which more often follow self-reported mental distress in males [58].

For age, we saw two competing trends: fewer recordings in patient registries (primary or specialist care) but more prescriptions for major depression with increasing age. Other studies have similarly found an increased use of antidepressants later in life [15]. When socio-economic status was measured by educational level we saw the same competing trends, with a lower proportion recorded in patient registries (primary or specialist care) for those with lower educational level, while this group was more often prescribed antidepressant drugs. For socio-economic status measured by income, this phenomenon was not present. Lower income was associated with a higher number of recordings in all central health registries. The clearer association between income and recordings in central health registries could be due to mental health problems affecting ability to hold a steady job. There was also a clear higher rate of being recorded in the prescription database in the HUNT area, suggesting that the use of drugs shows local variations [10].

### Using health registries to monitor depression in the population

In some ways, this study assumes that scores above cut-off in general population surveys represent the true prevalence of major depression. This assumption is certainly not fully valid. Firstly, not everybody will participate in such surveys. Depressed patients and people with low socioeconomic status are less likely to respond [21, 59]. Secondly, a high score on the HSCL and HADS scales may not represent a true major depression, even if we have chosen to use only those items most related to major depression. Short-term crises or events in life, that should not be seen or treated as major depression, could lead to high scores on these instruments.

### Strengths and limitations

In this study, we wanted to investigate the usefulness of central health registries in picking up major depression in the general population. This involves some challenges. Firstly, there has over time been a declining response rate in general population surveys. Previously, response rates could be above 80%, but in the current studies there was only a 65% attendance rate in the Tromsø studies [60] and around 54% in the HUNT studies [21]. Secondly, and related to this, there may be a selection bias, with those who have poorer health less likely to respond [61]. In this study, we found differences between sexes, age groups and across socio-economic status that could reflect such biases [11, 15, 48]. Thirdly, there may be reporting bias that differs between groups [62]. Fourthly, and related to the central health registries, we have demonstrated that far from all self-reported depressed patients were recorded in these the year following the survey. This could, in part, be due to under-treatment [36]. We opted to use certain diagnoses as outcomes (F32-34 from ICD-10 and ICPC P73 and P76 from ICPC), but arguments could be made for leaving out F34 and P73, as they are more related to bipolar disorder. However, there are limited instances of these diagnoses and it did not change the results if they were taken out. Also, we do not know how self-reported mental health problems are related to “the true” level of mental health problems, as the self-report instruments used are not diagnostic and may pick up on phenomena other than major depression. We saw, for example, that males and those with higher socio-economic status were less often recorded in the central health registries. Even if neither the population health surveys, nor the central health registries represent “a gold standard” for the prevalence of major depression in the population, this study still suggests some sort of stability over time in these registries. This makes it possible to use the central health registries to make estimates of the proportion of the population that will need treatment for mental health problems, and it makes it possible to track changes over time if other important assumptions do not change [63].

## Conclusion

In conclusion, this study - using data both from population self-report health surveys and central health registries - does not indicate any significant change in the average prevalence of major depression in the Norwegian population, but does suggest a change in the age distribution. The validity of self-report depression scoring instruments and data from central health registries align with previous research on the occurrence of depressive disorders.

The study illustrates a close relationship between score on either the HSCL or HADS scale in general population surveys and records in central health registries, be it in primary care, by filling prescriptions for antidepressants or in specialist care. Even if there is a low sensitivity of the registries and there is some group variation in how sensitive the central health registries are for picking up major depression, the stability of the measures over time indicates that following a combination of primary care data, prescription data and specialist care data from the central health registries may give a valid impression of the prevalence in the general population.

Combining data from population studies and central health registries could also have clinical implications. Our study highlighted the finding that people with depressive symptoms who have low educational level or who are older are more rarely recorded in central health registries, showing that they have fewer treatment consultations, even though they are more often treated with antidepressant drugs.

## Electronic supplementary material

Below is the link to the electronic supplementary material.

Supplementary Material 1

## Data Availability

The datasets used and/or analysed during this study are available from the corresponding author only after application has been made to and permission given by the ethics committee.

## Abbreviations

CPHR: Norwegian Control and Payment of Health Reimbursements Database – “the primary care database”
DALY: Disability-adjusted life year
HADS: Hospital Anxiety and Depression Scale
HSCL: Hopkins Symptom Checklist
HUNT: Trøndelag Health Study
ICD-10: International classification of disease version 10 (WHO)
ICPC-2: International classification of primary care (WHO)
NCD: Noncommunicable diseases
NorPD: Norwegian Prescription Database – “the prescription database”
NPR: Norwegian Patient Registry – “the specialist care registry”
SES: Socio-economic status
WHO: World Health Organization
