# Correction: The epidemiology of major depression among adults in Norway: an observational study on the concurrence between population surveys and registry data – a NCDNOR project

**DOI:** 10.1186/s12889-026-26272-0

**Published:** 2026-01-15

**Authors:** Jørgen Bramness, Vidar Hjellvik, Anne Høye, Martin Tesli, Marit Haram, Wenche Nystad, Steinar Krokstad

**Affiliations:** 1 Department of Alcohol, Tobacco and Drugs, Norwegian Institute of Public Health, P.O.Box 222, Oslo, 0213 Norway; 2 Norwegian National Advisory Unit on Concurrent Substance Abuse and Mental Health Disorders, Innlandet Hospital Trust, Brumunddal, Norway; 3 Department of Clinical Medicine, UiT The Arctic University of Norway, Tromsø, Norway; 4 Section for Clinical Addiction Research, Oslo University Hospital, Oslo, Norway; 5 Department of Chronic Diseases, Norwegian Institute of Public Health, Oslo, Norway; 6 Center for Clinical Documentation and Evaluation (SKDE), Tromsø, Norway; 7 Division of Mental Health and Substance Abuse, University Hospital of North Norway, Tromsø, Norway; 8 Department of Mental Health and Suicide, Norwegian Institute of Public Health, Oslo, Norway; 9 Division of Mental Health and Addiction, Oslo University Hospital, Oslo, Norway; 10 Department of Public Health and Nursing, Faculty of Medicine and Health Sciences, HUNT Research Centre, NTNU, Levanger, Norway; 11 Levanger Hospital, Nord-Trøndelag Hospital Trust, Levanger, Norway

**Correction: BMC Public Health 24**,** 1330 (2024)**

**https://doi.org/10.1186/s12889-024-18754-w**

Following publication of the original article [1], the authors would like to add the following reference under the Materials and methods section.

- Åsvold BO, Langhammer A, Rehn TA et al., Cohort profile update: The HUNT Study, Norway. Int J Epidemiol. 2023; 52(1):e80-e91. DOI: 10.1093/ije/dyac095.

The reference is inserted in the following statement as reference 37:

Both Tromsø surveys included adults 40–70 years of age and both HUNT surveys included adults 20–79 years of age [37].

The original article [1] has been updated.
