# Supplementary material for: The epidemiology of major depression among adults in Norway: an observational study on the concurrence between population surveys and registry data – a NCDNOR project
Source: BMC Public Health. 2024 May 17;24:1330. doi: 10.1186/s12889-024-18754-w (PMC11100182; doi:10.1186/s12889-024-18754-w)
Supplement: Supplementary file 1 — Supplementary Material 1 [file 12889_2024_18754_MOESM1_ESM.docx]

**Supplementary table S1.** Binary poisson regression analysis for the risk of being represented in central health registries (CPHR, NorPD, NPR or any). Data are given as relative risk (RR) and 95% robust confidence intervals for the RR. P-value for the RR can be found in table 5.

|  |  | Found in central health registries (unadjusted) | | | | | | | | Found in central health registries (adjusted ^a^) | | | | | | | |
| --- | --- | --- | --- | --- | --- | --- | --- | --- | --- | --- | --- | --- | --- | --- | --- | --- | --- |
|  |  | CPHR | | NorPD | | NPR | | Any | | CPHR | | NorPD | | NPR | | Any | |
|  | Ref. | RR | 95% CI | RR | 95% CI | RR | 95% CI | RR | 95% CI | RR | 95% CI | RR | 95% CI | RR | 95% CI | RR | 95% CI |
| **Tromsø7** |  |  |  |  |  |  |  |  |  |  |  |  |  |  |  |  |  |
| HSCL (above limit) | No | 8.46 | (7,10-10,07) | 6.14 | (5,11-7,38) | 11.86 | (8,52-16,52) | 7.23 | (6,30-8,29) | 6.79 | (5,64-8,16) | 5.48 | (4,53-6,64) | 10.10 | (7,17-14,23) | 6.10 | (5,29-7,04) |
| Age (10 years) | Cont. | 0.68 | (0,62-0,74) | 0.98 | (0,90-1,07) | 0.74 | (0,62-0,88) | 0.81 | (0,76-0,87) | 0.77 | (0,69-0,84) | 1.10 | (1,00-1,20) | 0.89 | (0,74- 1,08) | 0.91 | (0,85-0,98) |
| Sex | Male | 1.83 | (1,52-2,21) | 1.69 | (1,39-2,05) | 1.72 | (1,22-2,42) | 1.69 | (1,46-1,96) | 1.56 | (1,30-1,89) | 1.48 | (1,21-1,79) | 1.42 | (1,00- 2,01) | 1.47 | (1,27-1,70) |
| Education (medium) | High | 0.83 | (0,68-1,02) | 1.02 | (0,82-1,26) | 0.66 | (0,45-0,95) | 0.90 | (0,77-1,06) | 0.82 | (0,67-1,00) | 0.89 | (0,71-1,10) | 0.60 | (0,41- 0,88) | 0.84 | (0,71-0,99) |
| Education (low) | High | 0.91 | (0,70-1,18) | 1.54 | (1,21-1,97) | 0.72 | (0,44-1,19) | 1.23 | (1,01-1,49) | 0.78 | (0,60-1,02) | 1.15 | (0,90-1,47) | 0.57 | (0,34- 0,95) | 0.99 | (0,82-1,21) |
| Income, q3 | q4 | 1.42 | (1,06-1,90) | 1.34 | (1,00-1,79) | 1.51 | (0,90-2,54) | 1.39 | (1,11-1,75) | 1.28 | (0,96-1,71) | 1.31 | (0,98-1,76) | 1.41 | (0,84- 2,36) | 1.31 | (1,05-1,64) |
| Income, q2 | q4 | 2.35 | (1,78-3,09) | 2.08 | (1,57-2,74) | 2.21 | (1,34-3,64) | 2.21 | (1,78-2,74) | 1.91 | (1,45-2,51) | 1.83 | (1,37-2,42) | 1.80 | (1,09- 2,97) | 1.86 | (1,50-2,31) |
| Income, q1 | q4 | 3.84 | (2,90-5,08) | 3.14 | (2,36-4,19) | 3.65 | (2,18-6,10) | 3.47 | (2,79-4,33) | 2.52 | (1,88-3,37) | 2.20 | (1,63-2,98) | 2.42 | (1,43- 4,10) | 2.35 | (1,87-2,95) |
| **HUNT4** |  |  |  |  |  |  |  |  |  |  |  |  |  |  |  |  |  |
| HADS (7 above limit) | No | 4.66 | (4,24-5,13) | 3.56 | (3,25-3,89) | 6.90 | (5,64-8,45) | 3.82 | (3,56-4,10) | 4.54 | (4,12-5,01) | 3.43 | (3,13-3,76) | 6.82 | (5,54-8,39) | 3.68 | (3,42-3,95) |
| Age (10 years) | Cont. | 0.82 | (0,80-0,85) | 1.14 | (1,10-1,17) | 0.71 | (0,67-0,76) | 0.99 | (0,97-1,01) | 0.85 | (0,83-0,88) | 1.15 | (1,12-1,18) | 0.75 | (0,70-0,80) | 1.01 | (0,99-1,03) |
| Sex | Male | 1.89 | (1,69-2,10) | 1.86 | (1,68-2,05) | 1.77 | (1,41-2,21) | 1.85 | (1,71-2,00) | 1.86 | (1,67-2,07) | 2.03 | (1,84-2,23) | 1.63 | (1,30-2,05) | 1.94 | (1,79-2,09) |
| Education (medium) | High | 0.84 | (0,76-0,94) | 1.32 | (1,20-1,47) | 0.59 | (0,47-0,74) | 1.09 | (1,01-1,18) | 0.86 | (0,77-0,95) | 1.15 | (1,03-1,28) | 0.63 | (0,50-0,79) | 1.02 | (0,94-1,11) |
| Education (low) | High | 1.09 | (0,94-1,26) | 1.99 | (1,76-2,26) | 0.95 | (0,71-1,28) | 1.57 | (1,42-1,74) | 0.89 | (0,77-1,04) | 1.42 | (1,24-1,62) | 0.79 | (0,58-1,08) | 1.21 | (1,09-1,34) |
| Income, q3 | q4 | 1.16 | (0,99-1,35) | 1.12 | (0,97-1,28) | 1.21 | (0,88-1,65) | 1.15 | (1,02-1,28) | 1.13 | (0,97-1,31) | 1.08 | (0,94-1,24) | 1.17 | (0,86-1,59) | 1.11 | (1,00-1,25) |
| Income, q2 | q4 | 1.58 | (1,37-1,83) | 1.47 | (1,29-1,68) | 1.33 | (0,98-1,81) | 1.54 | (1,39-1,72) | 1.45 | (1,25-1,68) | 1.32 | (1,16-1,51) | 1.19 | (0,88-1,62) | 1.40 | (1,26-1,56) |
| Income, q1 | q4 | 2.07 | (1,77-2,41) | 2.05 | (1,79-2,36) | 1.91 | (1,39-2,63) | 2.09 | (1,87-2,33) | 1.68 | (1,44-1,96) | 1.63 | (1,41-1,88) | 1.48 | (1,08-2,05) | 1.68 | (1,50-1,88) |

a) adjusted for HSCL/HADS score, age (40-79 years of age in Tromsø and 20-79 years of age in HUNT), sex, educational level, and income. Abbreviations: CPHR: Norwegian Control and Payment of Health Reimbursements Database; NorPD: Norwegian Prescription Database; NPR: Norwegian Patient Registry; RR: Relative risk; HSCL: Hopkin’s Symptom Checklist; HADS: Hospital Anxiety and Depression Scale; q: quartile.

**Supplementary table S2.** Sensitivity and specificity for the central health registers when it comes to picking up depression in the population (a "true depression" is considered to be a score above the threshold in the population surveys).

|  |  | | Early study (Tromsø6 and HUNT3) | |  | Late study (Tromsø7 and HUNT4) | | | | | | | |
| --- | --- | --- | --- | --- | --- | --- | --- | --- | --- | --- | --- | --- | --- |
| Study area | Group | |  | |  |  | | | | | | | |
|  |  |  | CPHR | |  | CPHR | | NorPD | | NPR | | Any | |
|  |  |  | Sensitivity | Specificity |  | Sens | Spec | Sens | Spec | Sens | Spec | Sens | Spec |
| Tromsø | All | All | 0,11 (0,09-0,13) | 0.99 |  | 0,11 (0,10-0,13) | 0.99 | 0,09 (0,08-0,10) | 0.99 | 0,04 (0,03-0,05) | 1 | 0,16 (0,15-0,18) | 0.98 |
|  | Sex | Female | 0,12 (0,10-0,15) | 0.98 |  | 0,12 (0,11-0,14) | 0.98 | 0,09 (0,08-0,11) | 0.98 | 0,04 (0,03-0,05) | 1 | 0,17 (0,15-0,20) | 0.97 |
|  |  | Male | 0,08 (0,06-0,12) | 0.99 |  | 0,09 (0,07-0,11) | 0.99 | 0,08 (0,06-0,10) | 0.99 | 0,04 (0,03-0,05) | 1 | 0,14 (0,12-0,16) | 0.98 |
|  | Age (years) | 40-49 | 0,12 (0,09-0,17) | 0.98 |  | 0,13 (0,11-0,16) | 0.98 | 0,09 (0,07-0,11) | 0.99 | 0,05 (0,04-0,07) | 1 | 0,18 (0,16-0,21) | 0.98 |
|  |  | 50-59 | 0,13 (0,09-0,17) | 0.99 |  | 0,12 (0,10-0,15) | 0.98 | 0,08 (0,06-0,10) | 0.98 | 0,04 (0,03-0,05) | 1 | 0,16 (0,14-0,19) | 0.97 |
|  |  | 60-69 | 0,09 (0,06-0,13) | 0.99 |  | 0,08 (0,06-0,11) | 0.99 | 0,09 (0,07-0,12) | 0.98 | 0,03 (0,02-0,05) | 1 | 0,13 (0,10-0,17) | 0.98 |
|  |  | 70-79 | 0,08 (0,04-0,13) | 0.99 |  | 0,03 (0,01-0,06) | 0.99 | 0,11 (0,06-0,17) | 0.98 | 0,01 (0,00-0,05) | 1 | 0,11 (0,07-0,18) | 0.98 |
|  | Education | Low | 0,11 (0,07-0,15) | 0.99 |  | 0,09 (0,06-0,12) | 0.99 | 0,13 (0,10-0,17) | 0.98 | 0,02 (0,01-0,05) | 1 | 0,17 (0,14-0,21) | 0.97 |
|  |  | Medium | 0,09 (0,07-0,12) | 0.99 |  | 0,10 (0,08-0,12) | 0.99 | 0,07 (0,05-0,09) | 0.99 | 0,03 (0,02-0,04) | 1 | 0,14 (0,11-0,16) | 0.98 |
|  |  | High | 0,14 (0,11-0,19) | 0.99 |  | 0,13 (0,11-0,15) | 0.99 | 0,09 (0,07-0,11) | 0.99 | 0,05 (0,04-0,07) | 1 | 0,18 (0,15-0,20) | 0.98 |
|  | Income | q1 | 0,12 (0,09-0,17) | 0.98 |  | 0,14 (0,11-0,17) | 0.97 | 0,12 (0,09-0,15) | 0.98 | 0,05 (0,04-0,08) | 1 | 0,21 (0,18-0,25) | 0.96 |
|  |  | q2 | 0,12 (0,09-0,17) | 0.99 |  | 0,12 (0,10-0,15) | 0.98 | 0,09 (0,07-0,12) | 0.98 | 0,04 (0,03-0,06) | 1 | 0,18 (0,15-0,21) | 0.97 |
|  |  | q3 | 0,09 (0,06-0,13) | 0.99 |  | 0,11 (0,08-0,14) | 0.99 | 0,07 (0,05-0,09) | 0.99 | 0,04 (0,02-0,05) | 1 | 0,14 (0,11-0,17) | 0.98 |
|  |  | q4 | 0,09 (0,06-0,14) | 0.99 |  | 0,06 (0,04-0,09) | 0.99 | 0,06 (0,04-0,08) | 0.99 | 0,02 (0,01-0,04) | 1 | 0,10 (0,08-0,14) | 0.99 |
|  |  |  |  |  |  |  |  |  |  |  |  |  |  |
| HUNT | All | All | 0,12 (0,12-0,13) | 0.97 |  | 0,13 (0,12-0,14) | 0.97 | 0,13 (0,12-0,14) | 0.96 | 0,04 (0,03-0,04) | 0.99 | 0,21 (0,20-0,22) | 0.95 |
|  | Sex | Female | 0,16 (0,15-0,18) | 0.96 |  | 0,16 (0,15-0,18) | 0.97 | 0,16 (0,15-0,18) | 0.95 | 0,05 (0,04-0,06) | 0.99 | 0,26 (0,24-0,28) | 0.93 |
|  |  | Male | 0,09 (0,08-0,10) | 0.98 |  | 0,09 (0,08-0,10) | 0.98 | 0,09 (0,08-0,10) | 0.98 | 0,03 (0,02-0,03) | 1 | 0,15 (0,13-0,16) | 0.97 |
|  | Age (years) | 20-29 | 0,19 (0,14-0,26) | 0.97 |  | 0,17 (0,14-0,21) | 0.96 | 0,11 (0,09-0,14) | 0.98 | 0,06 (0,05-0,09) | 0.99 | 0,22 (0,18-0,26) | 0.95 |
|  |  | 30-39 | 0,19 (0,15-0,23) | 0.97 |  | 0,19 (0,16-0,22) | 0.96 | 0,10 (0,08-0,13) | 0.97 | 0,07 (0,05-0,09) | 0.99 | 0,23 (0,19-0,26) | 0.94 |
|  |  | 40-49 | 0,16 (0,13-0,18) | 0.97 |  | 0,16 (0,13-0,19) | 0.97 | 0,12 (0,10-0,14) | 0.97 | 0,05 (0,04-0,07) | 0.99 | 0,22 (0,19-0,25) | 0.95 |
|  |  | 50-59 | 0,15 (0,13-0,17) | 0.97 |  | 0,13 (0,11-0,15) | 0.97 | 0,12 (0,10-0,15) | 0.96 | 0,04 (0,03-0,05) | 0.99 | 0,21 (0,19-0,24) | 0.94 |
|  |  | 60-69 | 0,09 (0,08-0,11) | 0.98 |  | 0,11 (0,09-0,13) | 0.98 | 0,14 (0,12-0,16) | 0.96 | 0,02 (0,01-0,03) | 1 | 0,20 (0,18-0,22) | 0.95 |
|  |  | 70-79 | 0,07 (0,05-0,09) | 0.98 |  | 0,06 (0,05-0,08) | 0.98 | 0,16 (0,13-0,18) | 0.95 | 0,01 (0,00-0,01) | 1 | 0,18 (0,16-0,21) | 0.95 |
|  | Education | Low | 0,12 (0,10-0,13) | 0.96 |  | 0,12 (0,10-0,14) | 0.97 | 0,14 (0,12-0,17) | 0.94 | 0,03 (0,02-0,05) | 0.99 | 0,22 (0,20-0,25) | 0.92 |
|  |  | Medium | 0,12 (0,11-0,13) | 0.97 |  | 0,11 (0,10-0,12) | 0.97 | 0,13 (0,11-0,14) | 0.96 | 0,03 (0,02-0,03) | 1 | 0,19 (0,17-0,20) | 0.95 |
|  |  | High | 0,15 (0,13-0,17) | 0.97 |  | 0,16 (0,15-0,18) | 0.97 | 0,12 (0,11-0,14) | 0.97 | 0,06 (0,05-0,07) | 0.99 | 0,22 (0,20-0,24) | 0.95 |
|  | Income | q1 | 0,15 (0,13-0,17) | 0.96 |  | 0,15 (0,13-0,17) | 0.96 | 0,15 (0,13-0,17) | 0.95 | 0,04 (0,03-0,06) | 0.99 | 0,25 (0,23-0,27) | 0.92 |
|  |  | q2 | 0,12 (0,10-0,13) | 0.97 |  | 0,13 (0,11-0,14) | 0.97 | 0,14 (0,12-0,16) | 0.96 | 0,03 (0,03-0,04) | 0.99 | 0,21 (0,19-0,24) | 0.94 |
|  |  | q3 | 0,11 (0,09-0,13) | 0.97 |  | 0,12 (0,10-0,13) | 0.98 | 0,11 (0,09-0,13) | 0.97 | 0,04 (0,03-0,05) | 1 | 0,18 (0,16-0,20) | 0.95 |
|  |  | q4 | 0,12 (0,09-0,14) | 0.98 |  | 0,11 (0,09-0,13) | 0.98 | 0,11 (0,09-0,14) | 0.97 | 0,03 (0,02-0,04) | 1 | 0,17 (0,15-0,20) | 0.96 |

Abbreviations: CPHR: Norwegian Control and Payment of Health Reimbursements Database; NorPD: Norwegian Prescription Database; NPR: Norwegian Patient Registry; RR: Relative risk; HSCL: Hopkin’s Symptom Checklist; HADS: Hospital Anxiety and Depression Scale; q: quartile.

**Supplementary figure S1.** The left panels are graphical presentations of the number of respondents scoring above the cut-off in the early and late Tromsø (HSCL=1.85) and HUNT (HADS=7) surveys. Circles and bullets show proportions in 10-year age groups (20-29, 30-39, … 70-79). Dashed and solid lines show predicted proportions from a Poisson regression with age as a continuous variable (40-79 years in Tromsø and 20-79 years in HUNT). The right panels are similar graphical presentations of the share of those scoring above cut-off that are recorded in the CPHR primary care database within one year after the survey.
